# Decreased vitamin D levels in the pediatric population after COVID-19 lockdown

**DOI:** 10.1186/s13052-023-01515-7

**Published:** 2023-09-05

**Authors:** Paolo Cavarzere, Romina Pausilli, Lara Nicolussi Prinicpe, Rossella Gaudino, Alessandra Guzzo, Gaetano Cantalupo, Franco Antoniazzi

**Affiliations:** 1 Pediatric Division, Department of Pediatrics, University Hospital of Verona, Piazzale Stefani 1, 37126 Verona, Italy; 2 Department Surgical Sciences, Dentistry, Gynecology and Pediatrics, Pediatric Clinic, University of Verona, Verona, Italy; 3 Laboratory Unit, Department of Neurosciences, Biomedicine and Movement Sciences, University of Verona, Verona, Italy; 4 Innovation Biomedicine Section, Department of Engineering for Innovation Medicine, University of Verona, Verona, Italy; 5 Child Neuropsychiatry Unit, University Hospital of Verona, Verona, Italy; 6 Regional Center for the Diagnosis and Treatment of Children and Adolescents With Rare Skeletal Disorders. Pediatric Clinic, Department of Surgical Sciences, Dentistry, Gynecology and Pediatrics, University of Verona, Verona, Italy

## Abstract

**Backgroud:**

SARS-Cov2 infection began to spread worldwide since December 2019; on March 2020, the World Health Organization characterized its related disease, named COVID-19, as a pandemic. In Italy, to contain the spread of infection a severe lockdown in the spring 2020 was instituted. Other less severe restrictions were imposed in the winter 2020–2021 and in the spring 2021. The containment measures caused a series of consequences for the population and, in particular, for children and adolescents that presented psychophysical problems.

The aim of this manuscript is to investigate the serum levels of vitamin D in children and adolescents before, during and after the lockdown consequent to COVID-19 pandemic.

**Methods:**

This is a retrospective cross-sectional study, including all children and adolescents between 1 to 18 years referring to the Pediatric Endocrinology Service of the University Hospital of Verona, Italy, between January 2019 and December 2021. All patients affected by clinical conditions that involve vitamin D metabolism or assuming vitamin D supplementation were excluded.

**Results:**

In total, 491 children (36.7% males and 63.3% females) were enrolled in this study. The vitamin D levels decreased over time: 28.3 ± 10.2 ng/mL in 2019; 28.2 ± 11.4 ng/mL in 2020 and 24.9 ± 10.1 ng/mL in 2021 (*p* < *0.05*). The vitamin D levels are significant higher in summer and in autumn in comparison with the levels of winter and spring, regardless of the examined years.

**Conclusions:**

The measures adopted to contain the COVID-19 pandemic led to a reduction of serum levels of vitamin D in pediatric population, probably due to the reduced solar exposure. This may have severe consequences on the bone metabolism of those children who did not present optimal vitamin D levels even before the lockdown. Therefore, an adequate supplementation of vitamin D is necessary from the end of fall to the beginning of spring (November–April) in all children and adolescents living in Northern Italy.

## Backgroud

SARS-Cov2 infection and its related disease, named COVID-19, began to spread worldwide since December 2019 and, on March 2020, the World Health Organization characterized this disease as a pandemic [1]. Italy was the first European country with a high number of COVID-19 infections outside Asia, and, the state of sanitary emergency was proclaimed in January 2020, when government imposed the first containment measures to reduce the rate of virus transmission [2]. Due to the increase in the number of infections, the containment measures have been progressively tightened up, culminating in a severe lockdown from March 10^th^,2020 to May 4^th^,2020 [3]. As consequence of the lockdown, infections significantly decreased. This allowed a slow and progressive reduction of the restriction measures. In November 2020, due to a second wave of COVID-19, the Italian Government introduced new containment measures [4] and in our zone, namely Veneto region, a new lockdown was established for a period of 15 days in March 2021, although in milder form in comparison with the one of spring 2020. Successively, SARS-CoV2 infection remained widespread in the country, but thanks to the increasing use of more targeted containment measures (such as nasopharyngeal swab and/or the vaccination) other new severe lockdowns were avoided [5].

Although the containment measures, from the lockdown to the school closure and the ban for the outdoor activities, were necessary to reduce the rate of the infections transmission [6, 7], they caused some consequences for the psychophysical health of children and adolescents, exacerbating pre-existing health problems and social inequalities [8]. Among these, it is worth reporting the reduction of time spent in sports and outdoor activities and the increase in screen time and disturbed sleeping patterns [9], exacerbating the risk factors for weight gain, obesity and eating disorders [10]. Furthermore, one should not forget the emotional consequences of the lockdown period [11, 12]. Finally, the reduction in time spent outdoors and consequently the children’s exposure to sunlight may have reduced vitamin D levels and consequently worsened bone health, which is fundamental during the growth.

Therefore, this study aims to investigate the serum levels of vitamin D in children and adolescents before, during and after the lockdown consequent to COVID-19 pandemic.

## Patients and methods

### Patients

This is a retrospective cross-sectional study that enrolled all healthy children, between the age of 1 and 18 years old, referred to the Pediatric Endocrinology Division of the Hospital of Verona, Italy in the years 2019 (pre-Covid-19-restriction measures period), 2020 (Covid-19-restriction measures period) and 2021 (post-Covid-19-restriction measures period) for a suspected alteration in growth rate or in pubertal time.

Exclusion criteria were: clinical conditions that could affect vitamin D intake, absorption and metabolism, such as eating disorders, skeletal disorders or malabsorption disorders (i.e. inflammatory bowel disease); children taking vitamin D oral supplementation; children under one year of age; children of non-Caucasian ethnicity.

Evaluation included measurement of body weight, height, body-mass index (BMI, weight in kg divided by height in meters squared), and pubertal development according to the Tanner and Whitehouse method. For boys, testicular volume was measured using Prader’s orchidometer. Height was measured with a Harpenden stadiometer and expressed in cm.

All children underwent serum vitamin D measurement. According to The Endocrine Society’s clinical practice guidelines, “vitamin D status” was categorized as “sufficiency” (25-hydroxyvitamin ≥ 30 ng/mL), “insufficiency” (between 20 and 29 ng/mL), and “deficiency” (< 20 ng/mL) [13]. Data of the vitamin D levels were compared between the patients of the pre-Covid-19-restriction measures period, Covid-19-restriction measures period and post-Covid-19-restriction measures period, according to sex, pubertal status, body weight, BMI, and season. To evaluate seasonal variability, the pre-Covid-19/Covid-19/post Covid-19 restriction measures periods were divided into spring (March, April, May), summer (June, July, August), autumn (September, October, November), and winter (December, January, February).

## Methods

Serum vitamin D was measured by an immunochemiluminescent assay (LIAISON®, Diasorin; DiaSorin Ltd, Schiphol Rijk, The Netherlands).

The study was conducted in compliance with the terms of the Helsinki II Declaration. The Institutional Ethics Committee of the provinces of Verona and Rovigo, Italy, took note of the retrospective conduct of the study and approved it in order to publish the results. Written informed consent was obtained from the parents or the guardians of each patient.

### Statistical analysis

Statistical analysis was performed using IBM Corp 2017, IBM *SPSS* Statistics for Windows, Version *26.0*. Armonk, NY, USA. Normal distribution was assessed by Kolmogorov–Smirnov test. Descriptive data are expressed in numbers with frequency, median plus range, or mean ± standard deviation (SD), as appropriate. Categorical variables and continuous variables with a non-normal distribution were both compared between different groups using non-parametric methods (Mann–Whitney U test and Kruskal–Wallis test, for two or more groups, respectively). Relations between continuous variables were explored by means of bivariate Spearman correlation analysis. Statistical significance was set at *p* < *0.05*; all tests were two-sided. Multiple univariate analysis was used to identify interactions between age, sex, puberty, BMI, seasons, and vitamin D levels. To determine the relative contribution of all these different factors on vitamin D levels, backward linear regression was performed with vitamin D levels as the dependent variable and age, gender, weight, height, BMI, puberty, seasons, and year of analysis as independent variables.

## Results

In this study, we included 491 patients (36.7% males, 63.3% females) with a median age of 10.4 [1.2–18.4] years: 201 in 2019, 171 in 2020 and 119 in 2021. The 40.7% of the study population was prepubertal, the remaining 59.3% was pubertal.

Among all patients, 123 (25%) were enrolled in the winter season, 102 (20.8%) in the spring, 132 (26.9%) in the summer, and 134 (27.3%) in autumn.

The main characteristics of the children and adolescents of our cohort, in relation of the year of analysis, are presented in Table 1.

**Table 1:** Characteristics of the patients included in the study

	**2019 (*n* = *201*)**	**2020 (*n* = *171*)**	**2021 (*n* = *119*)**	**Total (*n* = *491*)**
Age (years)	10.8 [1.2–18]	10.1 [1.6–18.4]	10.3 [2.0–18.3]	10.4 [1.2–18.4]
Sex: Males/Females (%)	44.8/55.2	29.8/70.2	32.8/67.2	36.7/63.3
Body Weight (SDS)	-0.17 ± 2.1	-0.28 ± 1.88	0.09 ± 1.64	-0.14 ± 1.92
Height (SDS)	0.74 ± 9.69	0.09 ± 6.79	-0.02 ± 1.89	0.33 ± 7.44
BMI (SDS)	-0.05 ± 1.41	-0.19 ± 1.26	0.19 ± 1.44	-0.06 ± 1.37

**Fig. 1:**
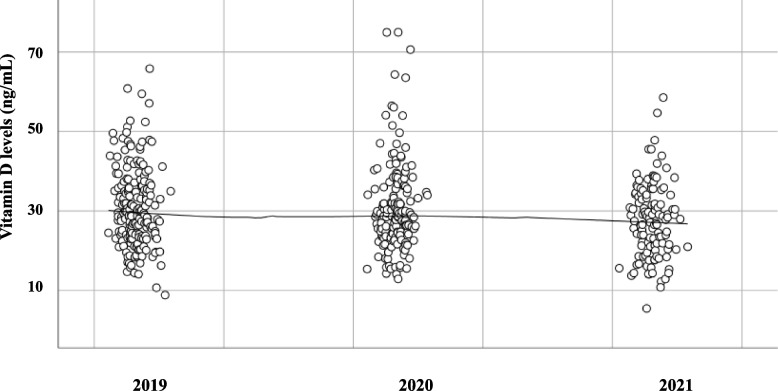
Vitamin D levels in relation to the year of determination. The regression line is shown

The vitamin D levels found in different years are represented in Fig. 1, documenting a significant difference of mean vitamin D level alongside the three years considered (*p* = *0.025*). The mean levels of vitamin D of our population in every year were insufficient: 28.3 ± 10.2 ng/mL in 2019, 28.2 ± 11.4 ng/mL in 2020 and 24.9 ± 10.1 ng/mL in 2021. In particular, in Table 2 the deficiency, insufficiency and sufficiency rates of vitamin D for year are reported. We identified a significant difference in distribution of different categories of “vitamin D status” (deficiency, sufficiency and insufficiency) across the different years (*p* = *0.039*) and seasons (*p* < *0.001*). In particular, pairwise comparisons revealed that the distribution of the three categories were significantly different in 2021 compared to both 2020 and 2019 (*p* = *0.031* and *p* = *0.017* respectively), while distribution was not significantly different between 2019 and 2020.

**Table 2:** The rates of  vitamin D during pre-Covid-19-restriction measures period, Covid-19-restriction measures period and post-Covid-19-restriction measures period

Vitamin D categories, n (%)	2019 (*n* = *201*)	2020 (*n* = *171*)	2021 (*n* = *119*)	Total (*n* = *491*)
Deficiency (≤ 20 ng/mL)	45 (22.5%)	40 (23.4%)	43 (36.1%)	128 (26.1%)
Insufficiency (20–29 ng/mL)	76 (38.0%)	74 (43.3%)	43 (36.1%)	193 (39.3%)
Sufficiency (≥ 30 ng/mL)	80 (39.5%)	57 (33.3%)	33 (27.8%)	170 (34.6%)

**Fig. 2:**
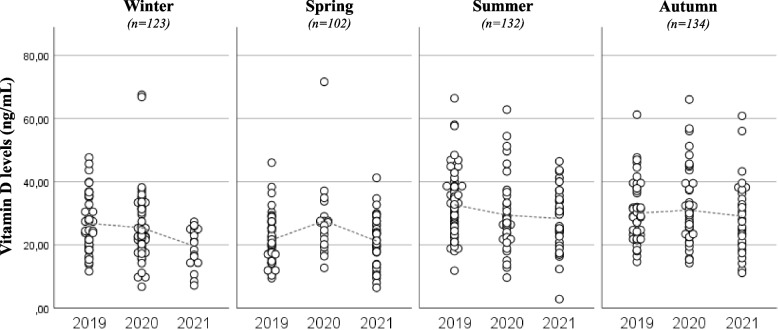
Vitamin D levels in relation to the years and the seasons. We identified a statistically significant difference between vitamin D levels of winter and spring and the vitamin D levels of summer and autumn, in all the years analyzed (*p* < *0.05*)

**Fig. 3:**
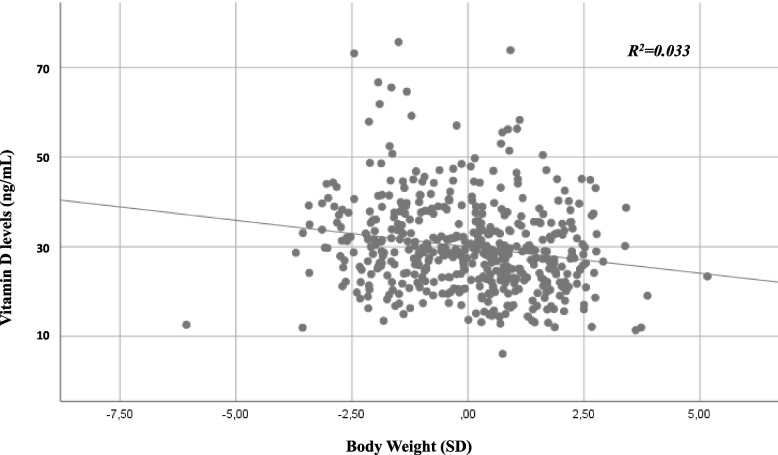
Vitamin D levels in relation to the body weight SD: we evidenced a reduction of vitamin D levels in relation to the increase of body weight SD. The regression line is shown (*p* < *0.05*)

Comparing the vitamin D levels between different seasons, we found significantly higher values in summer and in autumn in comparison with winter and spring, regardless of the examined years (*p* < *0.001*) (Fig. 2). Furthermore, we observed significant inverse correlations between vitamin D level and body weight SD (rho = -0.173; p < 0.001; Fig. 3), and BMI SD (rho = -0.165; p < 0.001). The backward linear regression revealed that only year (p = 0.027), season (p < 0.001) and body weight SD (p < 0.001), significantly contributed to the variance of vitamin D levels, net of other variables.

## Discussion

Our study allowed to observe that, in children and adolescents, vitamin D levels were reduced in post-pandemic period compared to previous levels, probably as a result of the restriction measures applied during and subsequently to the Covid-19 pandemic. To our knowledge, this is the first study that analyzed not only the vitamin D levels before and during the restriction measures, but also after these restrictions, in the pediatric population.

The first result that emerges from our analysis is that mean vitamin D level identified in children and adolescents of North-Eastern Italy might be already classified as insufficient before the restriction measures due to the Covid-19 pandemic. This result confirms previous data about vitamin D deficiency in different European and Italian areas. In fact, it is well known that in humans, the synthesis of vitamin D happens in the skin as a result of sunlight exposure. A recent survey estimate that 19% of children aged 4 to 10 years and 37% aged 11 to 18 years are vitamin D deficient by the end of the winter months and that circulating vitamin D is lowest from January to March for all children [14]. Moreover, despite relative abundance of sunlight availability in southern Europe and in the eastern Mediterranean area, the average prevalence of vitamin D concentration below 20 ng/mL ranges from 35 to 75% [15] and in Greece the prevalence of vitamin D concentration < 20 ng/mL among school children is 52.5% [16]. In our zone, a previous study identified a median vitamin D level of 21 ng/mL in a cohort characterized by Caucasian, African, North African and Indian children [17]. The higher vitamin D levels were found in Caucasians but were anyway lower than that highlighted in our population and however still insufficient. Similar data are reported in other different Italian cohorts, as shown by more recent research, conducted in children from center of Italy, without any selection or filtering process that described a mean serum vitamin D level of 28.2 ng/mL [17–19]. The cause of vitamin D insufficiency identified in our study is probably related to our zone, in the North-Eastern Italy (at 45° of latitude). An alteration in the zenith angle of the sun caused by a change in latitude, season of the year, or time of the day dramatically influences the skin's production of vitamin D and, above latitudes of approximately 33°, vitamin D synthesis in the skin decreases or even disappears during the winter time [20]. Recent data validate this affirmation showing the ineffectiveness of vitamin D production for at least one twelth of the year in regions between 23.5° and 66.5° latitude and, regarding Italy, the absence of vitamin D production from November to February as a result of sun exposure at the latitude of 43° [21]. Our better vitamin D levels, compared to the previous study by Franchi et al. carried out in Verona [17], are probably due to recently improved management of prophylaxis in the first year of life and to greater attention to this issue by pediatricians, especially for children at risk for vitamin D deficiency. In this context, it is useful to underline that all children enrolled in our cohort were of Caucasian origin, did not assume vitamin D supplementation, were older than one year of life, and did not present chronic diseases that can modify vitamin D levels. Consequently, our data are a realistic representation of healthy North-Eastern Italian children and adolescent population.

Our more intriguing finding is that the restriction measures and the prolonged home confinement subsequent to the Covid-19 pandemic are negatively correlated with vitamin D levels, regardless of gender, age, weight, puberty, and season. This is probably due to the prolonged reduction of sun exposure and consequently to a reduction in Vitamin D synthesis. In Italy in fact, as above mentioned, children are unable to synthesize vitamin D in the skin during late fall, winter months and early spring, even if sufficiently exposed to sunlight [22]. Thus, during this period an adequate vitamin D status can be maintained only from endogenous stores, accumulated during previous summer or by exogenous supplementation. Moreover, the food sources of vitamin D are poor and include egg yolks and oil-rich fish (such as salmon, mackerel, and herring). Therefore, the prolonged restriction measures with the school closures and the stop of recreational and sporting activities, reducing the sun exposure during springs of 2020 and during winter and spring 2021, resulted in further vitamin D deficiency at least in the pediatric population, with vitamin D levels in summer 2021 significantly reduced compared to the two previous summers.

Moreover, we found that the vitamin D levels were lower in 2021 compared to 2020. This finding is apparently the opposite to what one can expect, assuming that the restriction measures were more severe and prolonged in 2020 rather than in 2021. However, this might be due to the fact that the total time extent of the limitations – repeated intermittently for two consecutive years – reduced sun exposure with consequently vitamin D levels significantly lower. In fact, the consumption of the vitamin D stored in adipose and muscle tissues during the repeated implementation of restriction measures throughout the winter 2020–2021 and the subsequent spring, led to an additive reduction of the vitamin D levels in the following year.

Other factors that might influence the vitamin D levels, such as age, gender, BMI, puberty, and seasons were analyzed and were not found to be determinant factors in the reduction of vitamin D levels. Our result is more interesting because it takes into consideration not only the period of lock-down, as already described from other studies [23, 24], but also the period beyond the end of the restrictions. Therefore, while the government's restrictive measures have certainly avoided the spread of the COVID-19 pandemic, they also have reduced the levels of vitamin D, worsening bone health, which is fundamental during the growth, with consequences that we could only evaluate over time.

Another result underlined by our study is seasonal variability of the vitamin D levels, with lower levels in the winter and in the spring compared with summer and autumn, regardless of the examined years. It has already been described in many published studies [25, 26] and emphasizes once again the importance of the sun exposure for the vitamin D production. Vitamin D deficiency in fact is typical during the winter when children spend more time indoors, and this data is confirmed by our results.

Finally, our data support the relationship between body weight and vitamin D levels. Unlike other previous studies that described an inverse association of vitamin D levels and BMI greater than 30 kg/m^2^ [13, 27, 28], we found that vitamin D decreases as much as the body weight SD increases. This association is well known in children [29] and it might be caused by the characteristic of vitamin D that is fat soluble and consequently is trapped in adipocytes’ lipid droplets when stored. Consequently, in obese children there are low levels of vitamin D in blood despite the large amounts of vitamin D located in adipose tissue. Other hypotheses to explain this correlation concern the volumetric dilution and the negative feedback mechanisms from increased circulating 1,25-dihydroxyvitamin D levels [30]. Moreover, we can underline that the number of the obese children is progressively increasing over the years and that obesity may be a comorbidity of the prolonged lock-down as well as of reduction of vitamin D levels [31], so the pediatricians should pay particular attention to these categories of patients and advise them to supplement vitamin D.

In Italy, for healthy children and adolescents without risk factor for deficit of vitamin D a vitamin D prophylaxis is not suggested. Guidelines stress that first of all a healthy lifestyle associated with a normal BMI, including a healthy diet with vitamin D-containing foods and adequate outdoor activities, should be promoted in healthy children and adolescents [32]. A prophylaxis is instead recommended in conditions at risk for hypovitaminosis D: dark skin, inadequate sun exposure, international adoption, inadequate diets (i.e. vegan diet), chronic kidney disease, hepatic failure and/or cholestasis, malabsorption syndromes (i.e. cystic fibrosis, inflammatory bowel diseases, celiac disease at diagnosis), chronic therapies such as anticonvulsants, systemic glucocorticoids, antiretroviral therapy, systemic antifungals and obesity [33]. On the contrary, other societies systematically recommend vitamin D supplementation in children and adolescents during winter months or throughout the whole year, if reduced sun exposure is to be expected during the summer [34–36]. Considering the results of our studies and the reduction of sun exposition in relation to the change of life style consequent to Covid-19 pandemic, we suggest vitamin D supplementation from the end of fall to the beginning of spring (November–April) in all children and adolescents. For the doses of the prophylaxis, we recommend a range variable from 600 IU/day up to 1000 IU/day, following the indications of a recent Italian Consensus [33].

The present study is associated with some limitations: the most important is that it has a retrospective design, so we did not have data about nutritional intake and the physical activity, which are known to influence directly the vitamin D levels. Moreover, we did not compare the same population over the time, but we compare different population with well-matched characteristics (age, sex, BMI and pubertal development). Finally, another limitation of this study is the relatively small sample size, but the sample was homogeneous in the various years and in the different seasons.

## Conclusions

We demonstrated that in Italian children and adolescents, the restriction measures applied during and subsequently the Covid-19 pandemic favored a significant reduction in vitamin D levels, regardless of gender, age, weight, puberty and season; and that this reduction did not recover soon after the termination of the restrictive measures, but continued in the following months. In consideration of this data and of the fact that the mean vitamin D level identified in children and adolescents of North-Eastern Italy was already insufficient before the restriction measures, we suggest a vitamin D prophylaxis from the end of fall to the beginning of spring (November–April) in all children and adolescents. In this way, we intend to prevent the consequence of a prolonged vitamin D insufficiency in pediatric age.

## Data Availability

Not applicable.

## Abbreviations

BMI: Body Mass Index
SD: Standard Deviations
